# A Dynamical and Zero-Inflated Negative Binomial Regression Modelling of Malaria Incidence in Limpopo Province, South Africa

**DOI:** 10.3390/ijerph16112000

**Published:** 2019-06-05

**Authors:** Gbenga J. Abiodun, Olusola S. Makinde, Abiodun M. Adeola, Kevin Y. Njabo, Peter J. Witbooi, Ramses Djidjou-Demasse, Joel O. Botai

**Affiliations:** 1 Research Unit, Foundation for Professional Development, Pretoria 0040, South Africa; 2 Department of Mathematics and Applied Mathematics, University of the Western Cape, Private Bag X17, Bellville 7535, South Africa; pwitbooi@uwc.ac.za; 3 Department of Statistics, Federal University of Technology, Akure P.M.B 704, Nigeria; osmakinde@futa.edu.ng; 4 South African Weather Service, Private Bag X097, Pretoria 0001, South Africa; abiodun.adeola@weathersa.co.za (A.M.A.); joel.botai@weathersa.co.za (J.O.B.); 5 School of Health Systems and Public Health, Faculty of Health Sciences, University of Pretoria, Pretoria 0002, South Africa; 6 Institute of the Environment and Sustainability, University of California Los Angeles, Los Angeles, CA 90095, USA; kynjabo@ucla.edu; 7 MIVEGEC, IRD, CNRS, Univ. Montpellier, 34394 Montpellier, France; ramses.djidjoudemasse@ird.fr; 8 Department of Geography, Geoinformation and Meteorology, University of Pretoria, Private Bag X20, Hatfield 0028, South Africa

**Keywords:** malaria, climate, zero inflated negative binomial regression, dynamical models, Mopani, Vhembe

## Abstract

Recent studies have considered the connections between malaria incidence and climate variables using mathematical and statistical models. Some of the statistical models focused on time series approach based on Box–Jenkins methodology or on dynamic model. The latter approach allows for covariates different from its original lagged values, while the Box–Jenkins does not. In real situations, malaria incidence counts may turn up with many zero terms in the time series. Fitting time series model based on the Box–Jenkins approach and ARIMA may be spurious. In this study, a zero-inflated negative binomial regression model was formulated for fitting malaria incidence in Mopani and Vhembe―two of the epidemic district municipalities in Limpopo, South Africa. In particular, a zero-inflated negative binomial regression model was formulated for daily malaria counts as a function of some climate variables, with the aim of identifying the model that best predicts reported malaria cases. Results from this study show that daily rainfall amount and the average temperature at various lags have a significant influence on malaria incidence in the study areas. The significance of zero inflation on the malaria count was examined using the Vuong test and the result shows that zero-inflated negative binomial regression model fits the data better. A dynamical climate-based model was further used to investigate the population dynamics of mosquitoes over the two regions. Findings highlight the significant roles of *Anopheles arabiensis* on malaria transmission over the regions and suggest that vector control activities should be intense to eradicate malaria in Mopani and Vhembe districts. Although *An. arabiensis* has been identified as the major vector over these regions, our findings further suggest the presence of additional vectors transmitting malaria in the study regions. The findings from this study offer insight into climate-malaria incidence linkages over Limpopo province of South Africa.

## 1. Introduction

Malaria is a life-threatening disease that continues to claim a significant number of lives globally. In 2016 alone, malaria claimed roughly 445,000 lives across the globe from 216 million cases in 91 countries [1]. Despite various ongoing malaria control programmes, Africa continues to bear 90% of malaria cases and 91% of malaria deaths worldwide [1]. South Africa recently witnessed a significant increase in malaria cases across its epidemic regions, which are Limpopo, KwaZulu-Natal, and Mpumalanga province [2,3]. The sudden increase has been linked to climate and environmental factors [4], and reduction in indoor residual spraying [2]. In addition, the resurgence is more significant over Limpopo province. For instance, over 27,500 cases were reported in the province in 2017 as Mopani and Vhembe district municipalities presented the highest number of cases in the province [4,5]. *Anopheles arabiensis* has been identified as the major vector transmitting *Plasmodium falciparum* over the study regions [4,5].

Both malaria parasite and mosquito species are very sensitive to climatic conditions. Several studies [6,7,8,9,10,11,12] have investigated the impact of climate variables on the transmission of malaria and mosquito abundance. For instance, Craig et al. [13] developed a climate-based distribution model to investigate the impact of climate change on *An. gambiae* and malaria transmission over Sub-Saharan Africa. Hoshen and Morse [14] also developed a mathematical–biological model, comprising both the climate-dependent within-vector (*An. gambiae s.l.*) stages and the climate-independent within host stages to simulate malaria incidence in Zimbabwe. More recently, Abiodun et al. [12] developed mathematical models to investigate the impact of temperature and rainfall on the population dynamics of *An. arabiensis* malaria transmission over Nkomazi local municipality in KwaZulu-Natal province, South Africa. However, limited investigations have been made over Mopani and Vhembe districts; the regions in Limpopo province that are most prominent with respect to the malaria epidemic.

Recent studies have considered some statistical models for the transmission of malaria over some regions. For instance, various studies have presented various time series models based on the Box–Jenkins methodology [15,16,17]. Arab et al. [18] presented hierarchical Bayesian modelling of malaria in ten West African countries. Using Spearman correlation analysis, Adeola et al. [4] explored the roles of climate variables on malaria transmission in Mutale local municipality of Limpopo, South Africa. The analysis showed that monthly total rainfall, mean minimum temperature, mean maximum temperature, mean average temperature, and mean relative humidity were significantly and positively correlated with monthly malaria cases over the study areas. The monthly total rainfall and monthly mean minimum temperature came up as most significant. Malaria transmission is complex and involves a range of climatic, biological, and environmental factors. However, the high degree of non-linearity in these factors makes it difficult to predict and intervene against malaria [19]. Most statistical models are centred on time series approach grounded on the Box–Jenkins methodology [20]. The Box–Jenkins methodology has two approaches. These include the traditional autoregressive integrated moving average models and its seasonal extensions which do not allow for covariates different from lagged values of response variables. The other approach is the dynamic model (also referred to as ARIMAX), which allows for covariates different from its lagged values of the response variable [16]. Moreover, Briët et al. [21] formulated generalized seasonal autoregressive integrated moving average models for fitting monthly malaria case time series in a district in Sri Lanka, where malaria has decreased dramatically in recent years. In a real situation, malaria incidence counts may be inflated with many zeros. Fitting time series model based on the Box–Jenkins approach and ARIMA on malaria count data may give a spurious result. A zero-inflated model is designed to accommodate the extra zeros in the data.

Using a zero-inflated model for analysing malaria count data with an excessive number of zero, the present study investigates the impact of two climate variables on malaria incidence over Mopani and Vhembe. The malaria incidence is recorded in terms of the number of admission (number of inpatients) in all public health care stations in both regions. The zero-inflated negative binomial regression model was further developed to establish the links between climate variables and malaria cases over the study regions. In addition, the study simulates the population dynamics of *An. arabiensis* over both Mopani and Vhembe using climate-based mosquito model presented in the study of Abiodun et al. [8]. This is in order to investigate the impact of *An. arabiensis* abundance (in addition to climate) on malaria transmission over the epidemic regions.

## 2. Materials and Methods

### 2.1. Study Area

**Figure 1:**
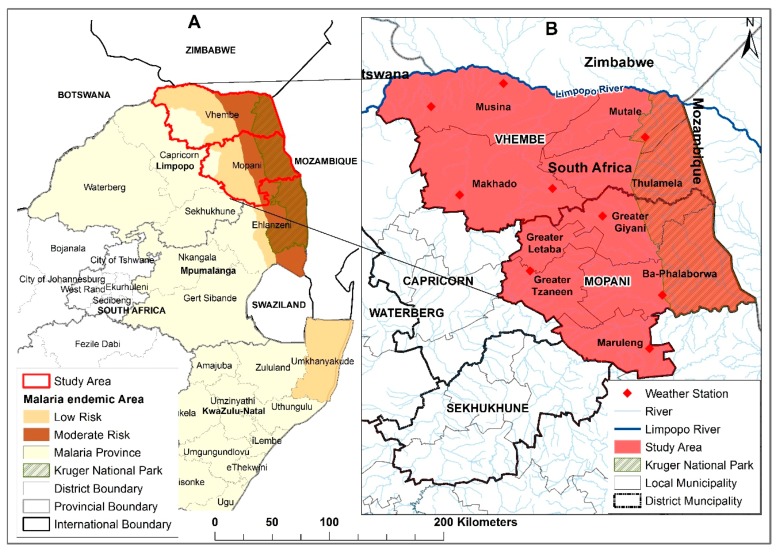
Location of study area, showing (**A**) the malaria risk classification of South Africa and Kruger National Park; (**B**) the location of weather stations, the local municipalities and Mopani and Vhembe district municipalities, Limpopo province, South Africa.

Vhembe and Mopani district municipalities are two of the five administrative district municipalities of Limpopo province, located in the north-eastern part of South Africa. The five district municipalities are further sub-divided into 25 local municipalities (Figure 1). According to the 2011 census, Limpopo province accommodates about 10% (5,404,868) of the total South African (51,770,560) population with 44.2% of the province’s population residing in Vhembe (24%) and Mopani (20.2%) districts [22]. These two districts account for about 96.3% of total malaria cases recorded within the province from 1998 to 2017, with 63.2% in Vhembe and 33.1% in Mopani. A large part of the study area is a remote area with pockets of commercial farms. The major malaria vector control strategies include the use of indoor residual spraying with Dichlorodiphenyltrichloroethane (DDT), larviciding of identified breeding habitats and insecticide-impregnated bed nets. Additionally, about 51% of the Kruger National Park, which records high malaria transmission is located within the study area [5]. The average annual temperature in both districts is 21.9 °C. In Vhembe an average of about 350 mm of rainfall is received while about 600 mm of rainfall is received in Mopani district.

### 2.2. Data

The malaria data reported in this study have been sourced from the provincial Integrated Malaria Information System (IMIS) of Malaria Control Programme in the Limpopo Provincial Department of Health and were obtained from the South African Weather Service (SAWS) through its collaborative research with the University of Pretoria Institute for Sustainable Malaria Control (UP ISMC), with ethical approval number MP_2014RP39_978. The data includes both active and passive surveillance malaria case patients, diagnosis date, sex, age, district and local council where the patient resides, source country or province in South Africa where the patient presumably contracted malaria and reported malaria deaths. The daily observation climatic data (total rainfall, maximum, minimum and mean temperatures) were also obtained from SAWS. The locations of the weather stations are shown in Figure 1. Both climate and malaria data span a period of 20 years (1 January 1998 to 31 December 2017).

### 2.3. Dealing with Missing Values

The malaria data denoted by M, consist of daily malaria incidence counts of Mopani and Vhembe District Municipalities from 1 January 1998 to 31 December 2017. The data were characterised by a large number of zeroes and some missing values. Predictor variables are the climate variables of the two districts: daily minimum temperature ($T_{t}^{min}$), daily maximum temperature ($T_{t}^{max}$) and daily total rainfall amount ($R_{t}$). For Mopani district, the proportions of the original data values that were missing are 0.00014, 0.00424 and 0.00424 for malaria count, daily minimum temperature and daily maximum temperature, respectively. For Vhembe district, the proportion of the original data values that were missing is 0.00096 for daily minimum temperature. In this study, multivariate imputation by chained equations (MICE) based on random forest was implemented for estimating a missing daily malaria count and missing values of some climate variables. Multivariate imputation by chained equations [23,24] estimate missing values for continuous data using predictive mean matching approach and binary data using logistic regression.

### 2.4. The Zero-Inflated Negative Binomial Regression Model

The effect of zero inflation on the malaria incidence is that the relationship may not be well-informed in terms of the significance of the correlation between malaria and some climate variables. For instance, the estimates of Spearman’s rank correlation coefficients between malaria and daily total rainfall of Mopani and Vhembe districts are 0.1342 (*p*-value < 2.2 × 10^−16^) and 0.1977 (*p*-value < 2.2 × 10^−16^) respectively. The measure of correlation between malaria and daily average temperature at lag 0 is 0.3001 (*p*-value < 2.2 × 10^−16^) for Mopani and 0.3754 (*p*-value < 2.2 × 10^−16^) for Vhembe. Similarly, measure of correlation between malaria and daily mosquito population at lag 0 is 0.0835 (*p*-value = 9.515 × 10^−13^) for Mopani and 0.1655 (*p*-value < 2.2 × 10^−16^) for Vhembe. The correlation values are very small but significant and show that daily rainfall, average temperature, and mosquito population do have a major influence on malaria prevalence in the two district municipalities of Limpopo, South Africa. The measure of the correlation between malaria count and each of the climate variables at lag 0 is significant but not significant in models for the district municipalities as shown in Table 1 and Table 2. The negative binomial distribution, also known as Poisson–Gamma mixture distribution is defined by its probability mass function as

$$P\left(y_{i}|\mu_{i},\;\theta \right)=\frac{\Gamma \left(y_{i}+\theta^{-1}\right)}{\Gamma \left(y_{i}+1\right)\Gamma \left(\theta^{-1}\right)}{\left(\frac{1}{1+\theta \mu_{i}}\right)}^{\theta^{-1}}{\left(\frac{\theta \mu_{i}}{1+\theta \mu_{i}}\right)}^{y_{i}}$$

where $\mu_{i}=t_{i}\mu$ and μ is the mean incidence rate of y per unit time $t_{i}$. Suppose a random variable $Y_{i}$ follows the negative binomial distribution. Then its conditional expected value is $E\left(Y_{i}|X_{i}\right)=E\left(\overset{p}{\underset{j=1}{\sum }}\beta_{j}X_{ji}\right)$ and the variance is $var\left(Y_{i}|X_{i}\right)=\mu_{i}+\theta \mu_{i}^{2}$, where θ is the over-dispersion parameter and $X_{1},\;X_{2},\;\ldots ,\;X_{p}$ are predictor variables.

Negative binomial regression is used to model count data with the condition that the variance of the data is much greater than its mean. As a result, it is very good for over-dispersed count data. Negative binomial regression model for count data expresses μ in terms of explanatory variables. It is assumed in this study that the dispersion parameter θ takes the same value at all predictor values, following [25].

Suppose that events $y_{1},\;y_{2},\;\ldots ,\;y_{n}$ are identically distributed. Then the probability distribution of the zero-inflated negative binomial random variable can be expressed as

$$y_{i}\sim \{\begin{array}{l} \;0\;with\;probability\;p_{i}\\ NB\left(\mu_{i},\theta \right)\;with\;probability\;1-p_{i}\; \end{array}$$

so that

$$y_{i}=\{\begin{array}{l} 0\;with\;probability\;p_{i}+\left(1-p_{i}\right){\left[\frac{1}{1+\theta \mu_{i}}\right]}^{\theta^{-1}}\\ \;y\;with\;probability\;\left(1-p_{i}\right)\left[\frac{\Gamma \left(y+\theta^{-1}\right){\left(\frac{1}{1+\theta \mu_{i}}\right)}^{\theta^{-1}}{\left(\frac{\theta \mu_{i}}{1+\theta \mu_{i}}\right)}^{y}}{\Gamma \left(\theta^{-1}\right)y!}\right]. \end{array}$$

A zero-inflated negative binomial (ZINB) regression model of the form:

$$\log\left(E\left(M_{t}|x_{i}\right)\right)=\beta_{0}+\overset{K}{\underset{k=9}{\sum }}\beta_{1k}R_{t-k}+\overset{K}{\underset{k=9}{\sum }}\beta_{2k}T_{t-k}^{ave}+\overset{K}{\underset{k=9}{\sum }}\beta_{3j}m_{t-j}$$

is formulated for malaria counts of Mopani and Vhembe districts, where $M_{t}$ denotes daily malaria count, $R_{t-k}$ denotes daily rain amount at lag k, $T_{t-k}^{ave}$ denotes daily average temperature at lag k and $m_{t-k}$ denotes simulated daily mosquito population at lag k. This model considers daily rain amount and its first *K* lagged values, average temperature and its first *K* lagged values and simulated daily mosquito population and its first *K* lagged values. It is assumed that patients are infected by mosquitoes before the day on which climate variables would be correlated with. It is noted that the incubation period of malaria within mosquito is 8–15 days depending on the daily temperature [26,27,28]. As a result, the value of K is taken to be 20.

It is observed that the time series structure makes malaria incidence counts dependent on each other. Ljung–Box test [29], a statistical test for determining whether any of a group of autocorrelations of a time series is different from zero, is employed to test if the residuals ($\eta_{t}$) of the zero-inflated negative binomial model ($M_{t}=E\left(M_{t}|x_{i}\right)+\;\eta_{t}$) are correlated. As a remedial measure, we suggest fitting a time series model based on autoregressive integrated moving average (ARIMA(p, d, q)) model to the residuals ($\eta_{t}$) of the fitted ZINB models following [30]. The ARIMA(p, d, q) on $\eta_{t}$ is defined as

$$\Delta^{d}\eta_{t}=\phi_{1}\Delta^{d}\eta_{t-1\;}+\phi_{2}\Delta^{d}\eta_{t-2\;}+\ldots +\phi_{p}\Delta^{d}\eta_{t-p\;}+\epsilon_{t\;}+\theta_{1}\epsilon_{t-1\;}+\theta_{2}\epsilon_{t-2\;}+\ldots +\theta_{q}\epsilon_{t-q\;}$$

where p, d and q are orders of autoregressive, integrated and moving average parts respectively. Residuals, $\epsilon_{t}$, of the fitted ARIMA model on $\eta_{t}$ are uncorrelated. The choice of optimal values of *p* and *q* are based on the ARIMA(p, d, q) model with the least Akaike information criterion and root mean square of error. The parameters of ARIMA model are estimated by minimising sum of square of $\epsilon_{t}$ using maximum likelihood estimation.

### 2.5. The Dynamical Mosquito Model

The importance of long-term data series in the analysis of climate impact on both mosquito abundance and malaria transmission have been highlighted in some studies [8,31,32]. However, long-term mosquito data are not easily accessible. For this reason, several studies [6,7,8,12] have used a deterministic model to simulate mosquito abundance over some regions. Similarly, due to the unavailability of mosquito data over the study regions, the present study used the dynamical model presented in the study of Abiodun et al. [8] to simulate abundance of *An. arabiensis* over Mopani and Vhembe. The climate-based model was developed to analyse how temperature and the availability of water affect mosquito population size. The model was validated over a town in eastern Sudan and was further used to investigate the influence of ambient temperature on the development and the mortality rate of *An. arabiensis* over Dondotha town in KwaZulu-Natal Province, South Africa. In particular, the model was used to examine the impact of climatic factors on the gonotrophic cycle and the dynamics of mosquito population over the study region. For details on the formulation of the mosquito model, we refer to Abiodun et al. [8].

The dynamical mosquito model was coded in MATLAB R2013b (MathWorks, Natick, MA, USA), while that of the regression model was handled by R programming language to implement methods in this paper. An R package *pscl* is used to implement zero-inflated negative binomial model, an R package *forecast* is used to implement autoregressive integrated moving average model and an R package *tseries* is used to implement Ljung–Box test and make plots of autocorrelation functions and partial autocorrelation functions.

## 3. Results and Discussion

### 3.1. Climate and Malaria Cases of Mopani and Vhembe

**Figure 2:**
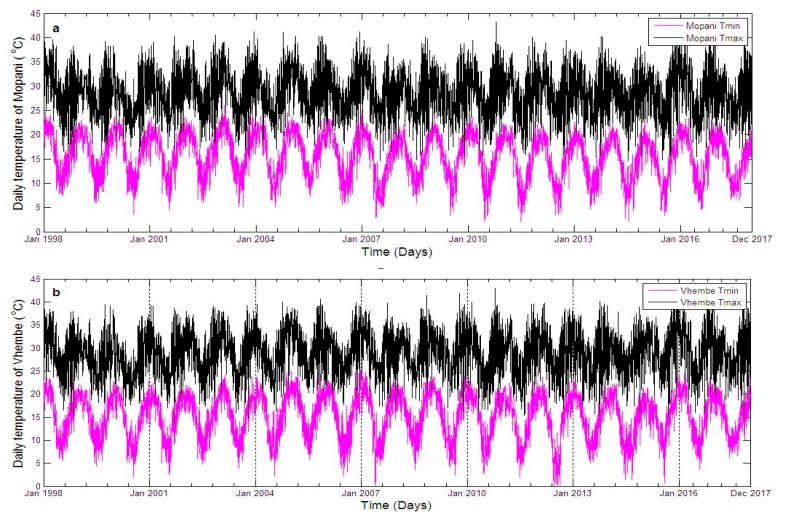
Time series of daily temperatures over calibration period showing the (**a**) daily maximum and minimum temperature of Mopani district municipality, (**b**) daily maximum and minimum temperature of Vhembe district municipality Limpopo province, South Africa from January 1998 to December 2017.

**Figure 3:**
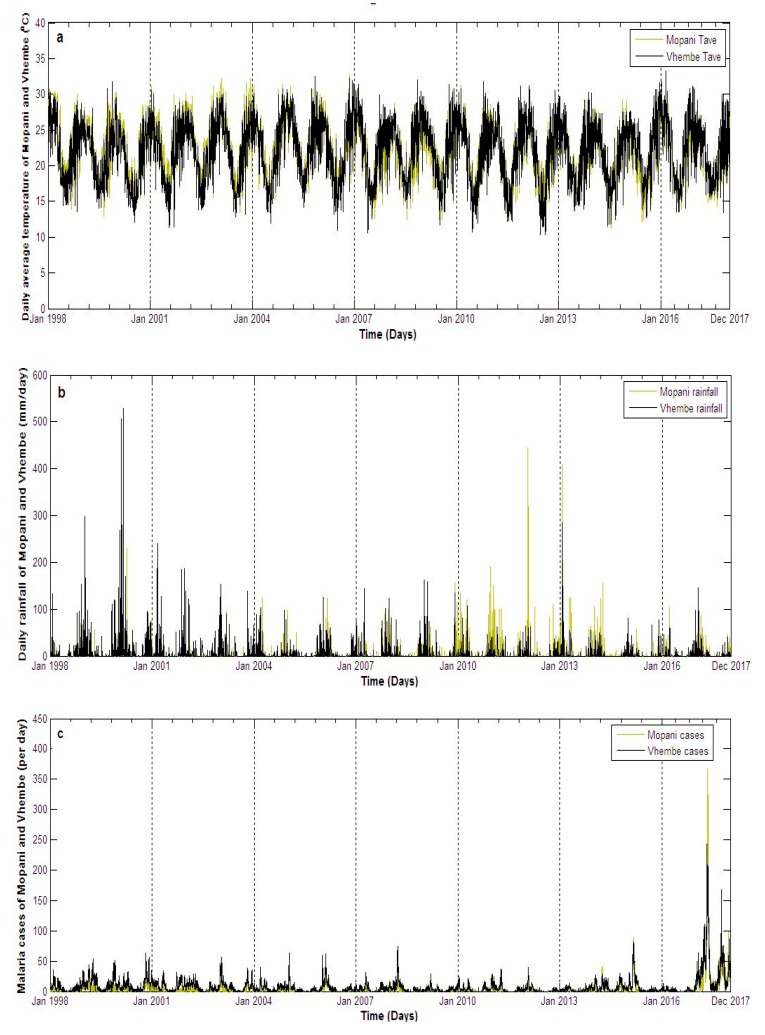
Time series of daily climate variables and malaria cases over calibration period comparing the (**a**) daily average temperature, (**b**) rainfall and (**c**) malaria cases of Mopani and Vhembe district municipality, Limpopo province, South Africa from January 1998 to December 2017.

Focussing on the study period (1 January 1998 to December 2017), results show that the daily maximum (black line) and minimum (pink line) temperature of Mopani fluctuates between 20–40 °C and 4–24 °C respectively, except for one day in January 2011 which is slightly above 40 °C (Figure 2a). Vhembe maximum and minimum temperature mainly fall between 20–40 °C and 3–24 °C respectively, except few days in January 2009, 2010 and 2011 which went above 40 °C for maximum and below 3 °C in June 2012 for minimum temperature (Figure 2b). The daily average temperature of Mopani falls within 15–30 °C with some variations of this range. For instance, the average temperature as high as 33 °C is observed around January of 2004, 2007, and 2016, and as low as 13 °C around July of 2007, 2010, 2011, 2012, 2014 and 2015 (Figure 3a). The daily average temperature of Vhembe fluctuates between 10 and 32 °C. Mopani rainfall is generally below 150 mm per day except in January of 2012 and 2013, which went up to roughly 420 mm and 400 mm per day respectively (Figure 3b). The rainfall pattern of Vhembe has decreased with time (Figure 3b). It was higher around 1999–2002 and lower from 2011–2017 except for some days in January 2013, which went as far as 300 mm/day from <100 mm/day on other days. New reported malaria cases over Mopani were normally below 100 per day but exceptionally high in 2017 (Figure 3c). The maximum cases of about 367 were recorded on the 4th of May 2017 as the early days of the month maintains 100 cases above. Malaria cases in Vhembe were also found below 100 cases/day except in 2017 that went far above this limit in April–May (Figure 3c) as the maximum cases hit 243 on the 26th of April 2017. The Mann–Kendall test can be employed to statistically assess if there is an upward or downward trend of average temperature and rain amount in the two districts over time. Using Mann–Kendall test, rainfall shows a statistically significant decreasing trends (*p*-value < 2.22 × 10^−16^) in both Mopani and Vhembe districts while daily temperature shows a significant decreasing trends (*p*-value < 2.22 × 10^−16^) in Mopani district and a non-significant decreasing trends (*p*-value = 0.92016) in Vhembe district over the study period.

Comparing the two study regions, Vhembe (in most days) seems hotter during the summer (December, January and February) months and cooler during the winter (June, July and August) months than Mopani although not statistically significant (*p*-value = 0.132). For instance, the black line (indicating Vhembe daily average temperature) is seen overlapping the green line (indicating Mopani daily average temperature) in most of the days (Figure 3a). However, the summer of Mopani was hotter than that of Vhembe in 2003 (*p*-value = 0.0033). The rainfall pattern shows that Vhembe generally experiences more rainfall than Mopani especially between 1998 and 2010 (*p*-value = 2.766 × 10^−6^) (Figure 3b). However, more rainfall is observed in Mopani than Vhembe between 2010 and 2014 (*p*-value = 3.614 × 10^−11^). Although similar patterns of malaria cases are observed over the two regions, the cases are more noticeable over Vhembe than Mopani (Figure 3c). One reason traceable to this could be that the climate variables of Vhembe are more conducive for malaria transmission than that of Mopani [4]. Malaria cases in both regions are also higher throughout 2017 compared to previous years, but the cases are slightly higher in Mopani than Vhembe in May 2017. The total malaria cases over the study period in Mopani and Vhembe are about 28,811 and 55,037 respectively. Following the 2011 census [33,34], the incident rate per 100,000 people in Mopani is calculated to be approximately 2637.15, while that of Vhembe is 4250.87. The Wilcoxon rank sum test with continuity correction is applied to test if daily rain amount, as well as daily average temperature and simulated daily mosquito abundance, of Mopani and Vhembe districts, is significantly different. The daily rain amount of Mopani and Vhembe districts are not statistically significantly different (*p*-value = 0.8803) over the study period. The daily average temperature of Mopani and Vhembe districts are not statistically significantly different (*p*-value = 0.6754) over the study period. The simulated daily mosquito abundance of Vhembe district is statistically higher than that of Mopani district (*p*-value = 0.0002) over the study period.

**Figure 4:**
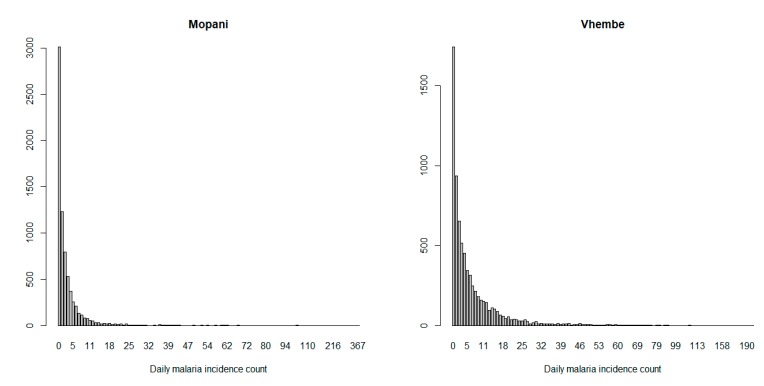
The barplot of malaria counts in Mopani and Vhembe district municipality, Limpopo province of South Africa.

Findings from the zero-inflated negative binomial regression model show that Mopani and Vhembe malaria incidence data are over-dispersed (Figure 4). This is because Mopani malaria count data has its variance (206.0995) greater than mean (4.0464). Similarly, Vhembe malaria count data has variance (201.0317) greater than mean (7.5342). Moreover, zero over-inflation of the malaria counts in both locations is evident in the figure as the number of days with no malaria count exceed the number of days with positive malaria count in each of the districts.

### 3.2. Analysis over Mopani District Municipality

A stepwise model selection procedure based on Akaike information criterion (AIC) was applied to drop models with highest AIC values in the fitted zero-inflated negative binomial model. The root mean square error (RMSE) of the full model for Mopani district, which is a measure of the deviation of observed malaria count from the fitted value, is 13.9049 while RMSE of the reduced model is 13.9137. The AIC value for the full model is 31,597.14, while the AIC value for the reduced model is 31,542.55. As a result, the reduced model is preferred for Mopani district.

The first block in Table 1 contains the count model coefficient and their standard error, *z*-score and *p*-value for each of the variables. The second block corresponds to the inflation model. The inflation model contains logit coefficients for predicting excess zeroes and the corresponding standard errors, *z*-scores and *p*-values for the coefficients. Table 1 presents the estimates of the zero-inflated negative binomial model (reduced model) for Mopani district. The coefficient of daily average temperature at lag 18 in the negative binomial regression part predicting the malaria count is statistically significant at 5% level of significance. The coefficients of daily rain amount at lag 9 and lag 16, daily average temperature at lag 9, lag 10, lag 12, lag 15 and lag 18, simulated daily mosquito population at lag 9, lag 10 and lag 20 in the logit model part predicting excessive zeroes are statistically significant. Other predictor variables are not statistically significant and are, therefore, excluded in the model. It is desirable to know whether zero-inflated negative binomial regression model fits the data statistically better than usual negative binomial regression model. The Vuong test [35] is employed to determine whether the formulated model (zero-inflated negative binomial regression model) fits the data better than the usual negative binomial regression model. The Vuong test is the likelihood-ratio-based test for model selection using the Kullback–Leibler information criterion. The test suggests that the zero-inflated negative binomial model is a significant improvement over a standard negative binomial model. The Vuong statistic tests the null hypothesis that the formulated zero-inflated negative binomial model and the negative binomial model are equally close to the true data generating process, against the alternative that the formulated zero-inflated negative binomial model is closer. The Vuong test is asymptotically distributed as a standard normal distribution (that is, N (0,1)) under the null hypothesis that the models are equivalent. The test rejects the null hypothesis at 5% level of significance (*p*-value < 2.22 × 10^−16^) and suggests that zero-inflated negative binomial model with lagged predictors fits the data better than the usual negative binomial regression model.

The number of malaria cases decreases by a factor of 0.9742 for a one-unit increase in daily average temperature at lag 18 when other variables are held constant. This implies that it is much likely to have any malaria cases as the daily average temperature at lag 9, lag 12 and lag 14 increase. The odds of being an excessive zero would decrease by 0.9404, 0.9335, 0.8481, 0.8872, 0.8668, 0.9242, 0.8729 and 0.9455 for every one-unit increase in daily rain amount at lag 9 and lag 16, daily average temperature at lag 9, lag 10, lag 12, lag 15 and lag 18, and simulated daily mosquito at lag 9 respectively. Increase in the odds of being an excessive zero means that it is less likely that there will be malaria cases. This implies that the likelihood that daily malaria count would be zero in Mopani district municipality decreases with an increase in daily rain amount at lag 9 and lag 16, daily average temperature at lag 9, lag 10, lag 12, lag 15 and lag 18, and simulated daily mosquito at lag 9. Moreover, the log odds of being an excessive zero would increase by 1.0366 and 1.0195 for every one-unit increase in the simulated daily mosquito at lag 10 and lag 20, respectively.

### 3.3. Analysis over Vhembe District Municipality

A stepwise model selection procedure based on Akaike information criterion (AIC) was applied to drop models with highest AIC values in the fitted zero-inflated negative binomial model for Vhembe district. The RMSE of the full model for Vhembe district is 13.7776 while RMSE of the reduced model is 13.79789. The AIC value for the full model is 42,218.47, while the AIC value for the reduced model is 42,232.6. As a result, the reduced model is preferred for Vhembe district.

Table 2 presents the estimates of the zero-inflated negative binomial model (reduced model) for Vhembe district. The coefficients of daily average temperature at lag 9, lag 12 and lag 14, simulated daily mosquito population at lag 20 in the count model predicting daily malaria count are statistically significant at 5% level of significance. The coefficients of daily average temperature at lag 10, lag 12 and lag 14, and simulated daily mosquito population at lag 9 and lag 15 in the logit model part predicting excessive zeroes are statistically significant. Other predictors are not statistically significant and are therefore excluded from the model. The Vuong test is also employed to determine whether a negative binomial regression model fits the Vhembe district malaria data statistically better than the formulated zero-inflated negative binomial regression model. The test rejects the null hypothesis at 5% level of significance (*p*-value < 2.22 × 10^−16^) and suggests that zero-inflated negative binomial regression model fits the data better than the negative binomial regression model.

The number of malaria cases increases by 1.0247, 1.0189 and 1.0151 for a one-unit increase in daily average temperature at lag 9, lag 12 and lag 14, respectively, when other variables are held constant. This implies that it is more likely to have any malaria cases as the daily average temperature at lag 9, lag 12 and lag 14 increase. The number of malaria cases decreases by a factor of 0.9979 for a one-unit increase in simulated daily mosquito population at lag 20 when other variables are held constant. This implies that it is less likely to have any malaria cases as the daily average temperature at lag 18 increase. The odds of being an excessive zero would decrease by 0.7965, 0.8848, 0.8364 and 0.9541 for every one-unit increase in daily average temperature at lag 10, daily average temperature at lag 12, daily average temperature at lag 14 and simulated daily mosquito population at lag 9 respectively. This implies that the likelihood that daily malaria count would be zero in Vhembe district municipality decreases with an increase in daily average temperature at lag 10, daily average temperature at lag 12, daily average temperature at lag 14 and simulated daily mosquito population at lag 9. Moreover, the odds of being an excessive zero would increase by a factor of 1.0296 for every one-unit increase in the simulated daily mosquito population at lag 15.

The dispersion parameter θ in Table 1 and Table 2 gives an indication if zero-inflated negative binomial model is fit for the data. If θ approaches infinity, then variance equals mean and as a result, zero-inflated Poisson model will fit the data better. Additionally, θ is finite implies that the variance is greater than mean. As θ approaches 0, the farther the variance is from the mean. Exponentiating log(θ) in Table 1 and Table 2, the values of θ are 0.4292 and 0.6257 for Mopani and Vhembe districts. Hence, the zero-inflated negative binomial model is appropriate for the model and confirm the result in Section 3.1.

This complements the findings of previous studies. It was argued in [36] that a moderate transmission intensity climate is crucial to malaria transmission. Based on the findings of [37,38] concluded that climate predictor variables generate a better predictive power when modelling malaria incidence in areas with unstable transmission compared to areas with stable endemicity. However, [36] shows that the development of clinical immunity buffers any effect of climate under high endemicity. In addition, [18] showed that there is a statistically significant correspondence between malaria rates and the climate variables, mostly air temperature and precipitation. This is confirmed in the fitted models for malaria incidence in Mopani and Vhembe districts. An increase in daily average temperature and its lagged values significantly raise the chance of malaria transmission and thereby leads to an increase in malaria incidence in Vhembe district. Furthermore, an increase in rainfall amount at lags 9 and 16 increases the probability of malaria cases occurring in the Mopani district. This is in line with several other studies that have highlighted the importance of rainfall on malaria transmission and other infectious diseases in western Kenya [39], Tanzania [40], East Africa [41] and Ghana [42].

**Figure 5:**
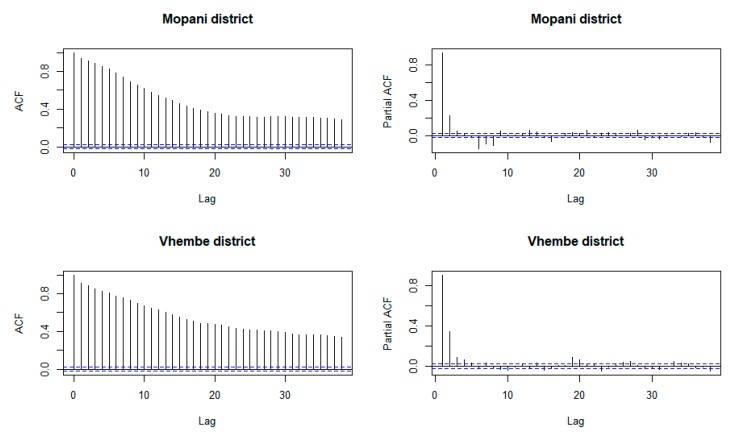
Plot of autocorrelation function and partial autocorrelation function of residuals of ZINB model for Mopani and Vhembe district municipalities.

Ljung–Box test [29] is employed to test if the residuals ($\eta_{t}$) of the zero-inflated negative binomial model ($M_{t}=E\left(M_{t}|x_{i}\right)+\;\eta_{t}$) are correlated. The Ljung–Box test shows that residuals of a fitted model for each of Mopani district (*p*-value < 2.2 × 10^−16^) and Vhembe district (*p*-value < 2.2 × 10^−16^) are autocorrelated. This confirms the result of plots of the autocorrelation function and partial autocorrelation function in Figure 5. The $\eta_{t}$ achieves stationarity at d=1. The optimal models for $\eta_{t}$ are ARIMA(5,1,4) and ARIMA(2,1,1) for Mopani and Vhembe district municipalities, respectively. The estimate of ARIMA(5,1,4) model for Mopani district are $\phi_{1}=-0.1991$, $\phi_{2}=0.5385$, $\phi_{3}=\;-0.2459$, $\phi_{4}=\;-0.2614$, $\phi_{5}=0.0716$, $\theta_{1}=-0.0854$, $\theta_{2}=-0.6160$, $\theta_{3}=0.4068$ and $\theta_{4}=0.2872$. The estimates of ARIMA(2,1,1) model for Vhembe district are $\phi_{1}=0.1252$, $\phi_{2}=0.0915$ and $\theta_{1}=-0.5565$.

**Figure 6:**
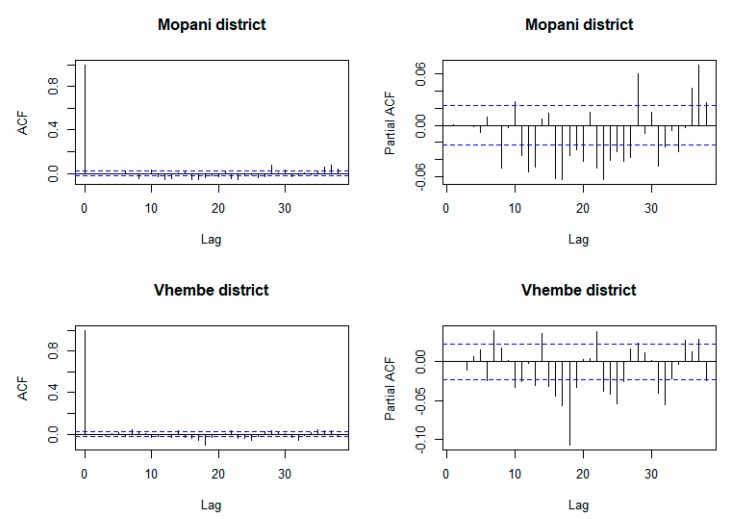
Plot of autocorrelation function and partial autocorrelation function of residuals of ZINB+ARIMA model for Mopani and Vhembe district municipalities.

**Figure 7:**
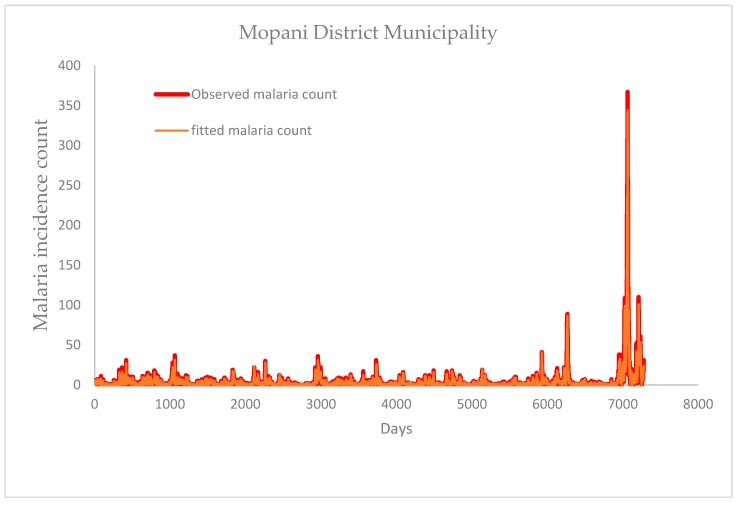
Comparison between fitted and observed malaria count for Mopani District.

**Figure 8:**
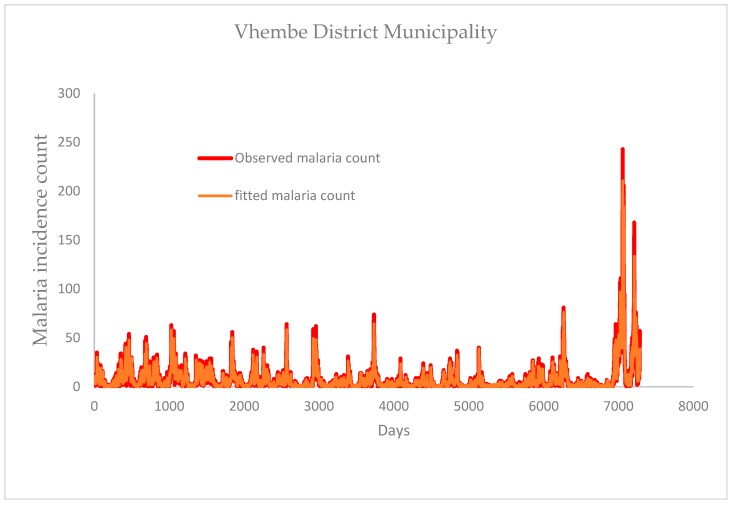
Comparison between fitted and observed malaria count for Vhembe District.

Figure 6 presents the correlograms of the autocorrelation function and partial autocorrelation function on the residuals of ZINB+ARIMA model on malaria incidence counts. The figure shows that residuals of the fitted ZINB+ARIMA model are not correlated. The Ljung–Box test confirms that the residuals of models for Mopani district (*p*-value = 0.9946) and Vhembe district (*p*-value = 0.9477) are not correlated. Figure 7 and Figure 8 present the comparison between the observed and fitted malaria counts over Mopani and Vhembe, respectively.

### 3.4. Mosquito Abundance and Malaria Cases of Mopani and Vhembe

**Figure 9:**
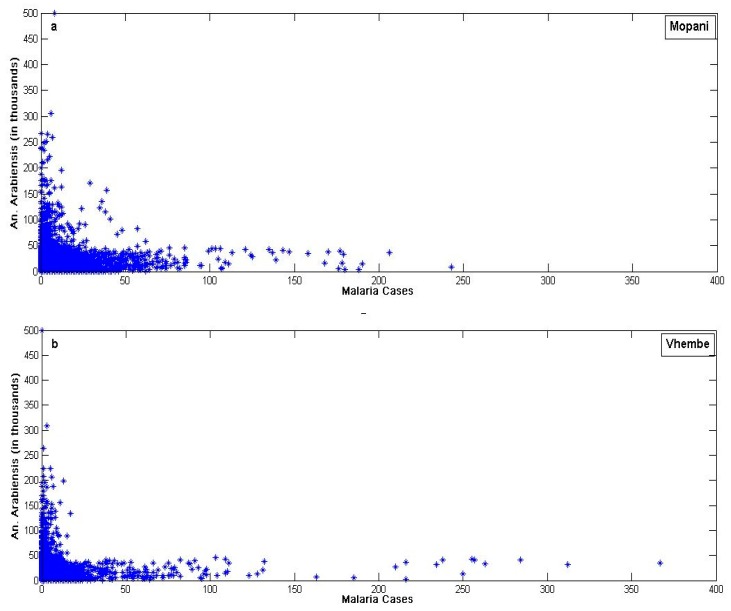
Scatter plot of daily simulated *An. arabiensis* and malaria cases over (**a**) Mopani and (**b**) Vhembe municipality, Limpopo province, South Africa from January 1998 to December 2017.

Findings further highlight the importance of mosquitoes in the transmission of malaria (Figure 9). Results also show that abundance of *An. arabiensis* is positively correlated with malaria transmission over the two study regions (Figure 9). The measure of the correlation (Spearman’s rank correlation coefficients) between mosquito abundance and malaria count is 0.0835 (*p*-value = 9.515 × 10^−13^) in Mopani district while the measure of the correlation between mosquito abundance and malaria count is 0.1655 (*p*-value < 2.2 ×10^−16^) in Vhembe district. However, findings show that transmission is possible over the study regions even with temperate amount of *An. arabiensis*. For instance, over Mopani, malaria cases maintain a steady increase from 0 to almost 250 even below estimated 60,000 *An. arabiensis* (Figure 9a). Similarly, with just about 50,000 *An. arabiensis,* malaria cases went up to 350 in Vhembe (Figure 9b). This is also an indication that the impact of other malaria vectors over the study regions cannot be overlooked. In other words, all control measures to eradicate malaria over these regions should target *An. arabiensis* and other malaria-transmitting vectors. Although it has been established that *An. arabiensis* is the primary malaria vector in South Africa [32], the findings here suspect the presence of additional mosquito species transmitting malaria over the study regions as recently found in KwaZulu-Natal and Mpumalanga province [32]. This is also in line with the findings of [5] where several other mosquito species were found across five different regions in Limpopo province [5].

## 4. Conclusions

In this study, the importance of climate variables on population dynamics of *An. arabiensis* and malaria transmission over Mopani and Vhembe (two epidemic regions in Limpopo) was investigated. In particular, a zero-inflated negative binomial regression model was formulated for predicting daily counts of malaria incidence in the two regions as a function of these variables. Results from the study show that daily average temperature, rain amount and simulated daily mosquito population at various lags affects the probability of having malaria count in the Mopani and Vhembe district municipalities. The time series structure of the data from the two district municipalities makes each of the malaria incidence count, simulated daily mosquito population and climate variables autocorrelated. Time series models based on autoregressive integrated moving average (ARIMA) are employed on the residuals of zero-inflated negative binomial models as a remedial measure. This gives better predictive models and the associated residuals are not autocorrelated, as supported by the Ljung–Box test.

In general, since there are no exceptional variations in the climate variables in 2017 (for example, daily average temperature (*p*-value = 0.0868), daily rain amount (*p*-value = 0.0867)), the sudden increase of the cases might not totally depend on climate. There could be other factors associated with the increase around this period. It could also be that malaria control activities were relaxed during this period as suggested by the National Institute for Communicable Diseases (NICD) (NICD update, 2017). A further reason could be that malaria transmission started in more areas in both study regions.

Due to unavailability of actual mosquito data over the study regions, the present study considered simulated mosquito data for its analyses. It is envisaged that actual data would produce more precise results in this type of study.

## Figures and Tables

**Table 1:** Estimates of zero-inflated negative binomial regression model for Mopani district municipality.

Count Model Coefficients (Negbin with Log Link):					
	Estimate	Std. Error	*z* Value	Pr (>|z|)	Confidence Interval
Intercept	3.169	0.1849	17.136	<2 × 10^−16^ ***	(2.8066, 3.5314)
Daily average temperature at lag 18	−0.0261	0.0085	−3.074	0.0021 **	(−0.0428, −0.0094)
Log(theta)	−0.8459	0.0254	−33.296	<2 × 10^−16^ ***	(−0.8957, −0.7961)
**Zero-Inflation Model Coefficients (Binomial with Logit Link):**					
	**Estimate**	**Std. Error**	***z* value**	**Pr (>|z|)**	**Confidence Interval**
Intercept	11.2884	0.7138	15.814	<2 × 10^−16^ ***	(9.8894, 12.6874)
Daily rain amount at lag 9	−0.0614	0.0297	−2.072	0.0383 *	(−0.1196, −0.0032)
Daily rain amount at lag 16	−0.0688	0.0325	−2.118	0.0342 *	(−0.1325, −0.0051)
Daily average temperature at lag 9	−0.1648	0.0428	−3.852	0.000117 ***	(−0.2487, −0.0809)
Daily average temperature at lag 10	−0.1197	0.0464	−2.578	0.0099 **	(−0.2106, −0.0288)
Daily average temperature at lag 12	−0.1430	0.0328	−4.356	1.32 × 10^−5^ ***	(−0.2073, −0.0787)
Daily average temperature at lag 15	−0.0788	0.0301	−2.623	0.0087 **	(−0.1378, −0.0198)
Daily average temperature at lag 18	−0.1359	0.033	−4.124	3.73 × 10^−5^ ***	(−0.2006, −0.0712)
Simulated daily mosquito population at lag 9	−0.056	0.0091	−6.188	6.10 × 10^−10^ ***	(−0.0738, −0.0382)
Simulated daily mosquito population at lag 10	0.0359	0.0067	5.385	7.26 × 10^−8^ ***	(0.0228, 0.0490)
Simulated daily mosquito population at lag 20	0.0193	0.0048	4.032	5.53 × 10^−5^ ***	(0.0099, 0.0287)

Signif. codes: 0 ‘***’ 0.001 ‘**’ 0.01 ‘*’ 0.05 ‘.’ 0.1 ‘ ’ 1; Theta 0.4292; Number of iterations in BFGS optimization: 1; Log-likelihood: −1.575 × 10^4^ on 23 Df.

**Table 2:** Estimates of zero-inflated negative binomial regression model for Vhembe district municipality.

Count Model Coefficients (Negbin with Log Link):					
	Estimate	Std. Error	*z* Value	Pr (>|z|)	Confidence Interval
Intercept	0.8355	0.1406	5.941	2.84 ×10^−9^ ***	(0.5599, 1.1112)
Daily average temperature at lag 9	0.0244	0.0083	2.924	0.00346 **	(0.0080, 0.0407)
Daily average temperature at lag 12	0.0187	0.0072	2.598	0.00939 **	(0.0046, 0.0328)
Daily average temperature at lag 14	0.0150	0.0067	2.248	0.02460 *	(0.0019, 0.0281)
Simulated daily mosquito population at lag 20	−0.0021	0.0009	−2.361	0.01820 *	(−0.0039, −0.0004)
Log(theta)	−0.4689	0.0217	−21.616	<2 × 10^−16^ ***	(−0.5115, −0.4264)
**Zero-Inflation Model Coefficients (Binomial with Logit Link):**					
	**Estimate**	**Std. Error**	***z* value**	**Pr (>|z|)**	**Confidence Interval**
Intercept	9.6683	0.7061	13.692	<2 × 10^−16^ ***	(8.2843, 11.0523)
Daily average temperature at lag 10	−0.2275	0.05441	−4.186	2.85 × 10^−5^ ***	(−0.3340, −0.1210)
Daily average temperature at lag 12	−0.1224	0.04241	−2.886	0.003896 **	(−0.2055, −0.0393)
Daily average temperature at lag 14	−0.1787	0.0417	−4.282	1.85 × 10^−5^ ***	(−0.2606, −0.0969)
Simulated daily mosquito population at lag 9	−0.0470	0.0124	−3.784	0.000154 ***	(−0.0713, −0.0226)
Simulated daily mosquito population at lag 15	0.0292	0.0059	4.986	6.16 × 10^−7^ ***	(0.0177, 0.0407)

Signif. codes: 0 ‘***’ 0.001 ‘**’ 0.01 ‘*’ 0.05 ‘.’ 0.1 ‘ ’ 1; Theta 0.6257; Number of iterations in BFGS optimization: 1; Log-likelihood: −2.11 × 10^4^ on 17 Df.

## Data Availability

The malaria data reported in this manuscript have been sourced from the provincial Integrated Malaria Information System (IMIS) of malaria control programme in the Mpumalanga Provincial Department of Health and was obtained from the South African Weather Service (SAWS) through its collaborative research with the University of Pretoria Institute for Sustainable Malaria Control (UP ISMC). The climate data were obtained from the National Center for Environmental Prediction (NCEP) and Climate Forecast System Reanalysis (CFSR), and the Tropical Rainfall Measuring Mission (TRMM).

## References

[1] World Health Organization (2018). WHO Update, World Malaria Report. http://www.who.int/mediacentre/factsheets/fs094/en/.

[2] National Institute for Communicable Diseases (NICD) (2017). Update. http://www.nicd.ac.za/wp-content/uploads/2017/05/Malaria-update.pdf.

[3] Abiodun G.J., Maharaj R., Witbooi P., Okosun K.O. (2016). Modelling the Influence of Temperature and Rainfall on the Population Dynamics of Anopheles Arabiensis. Malar. J..

[4] Adeola A.M., Botai J.O., Rautenbach H., Adisa O.M., Ncongwane K.P., Botai C.M., Adebayo-Ojo T.C. (2017). Climatic Variables and Malaria Morbidity in Mutale Local Municipality, South Africa: A 19-Year Data Analysis. Int. J. Environ. Res. Public Health.

[5] Munhenga G., Brooke B.D., Spillings B., Essop L., Hunt R.H., Midzi S., Govender D., Braack L., Koekemoer L.L. (2014). Field study site selection, species abundance and monthly distribution of Anopheline mosquitoes in the northern Kruger National Park, South Africa. Malar. J..

[6] Ermert V., Fink A.H., Morse A.P., Paeth H. (2012). The Impact of Regional Climate Change on Malaria Risk Due to Greenhouse Forcing and Land-Use Changes in Tropical Africa. Environ. Health Perspect..

[7] Tompkins A.M., Ermert V. (2013). A regional-scale, high resolution dynamical malaria model that accounts for population density, climate and surface hydrology. Malar. J..

[8] Abiodun G.J., Witbooi P., Okosun K.O. (2017). Mathematical modelling and analysis of mosquito-human malaria model. Int. J. Ecol. Econom. Stat..

[9] Abiodun G.J. (2017). A Mathematical Model for Studying the Impact of Climate Variability on Malaria Epidemics in South Africa. Ph.D. Thesis.

[10] Abiodun G.J., Witbooi P., Okosun K.O. (2017). Modelling and analysing the impact of temperature and rainfall on mosquito population dynamics over KwaZulu-Natal Province, South Africa. Int. J. Biomath..

[11] Abiodun G.J., Witbooi P., Okosun K.O. (2018). Modelling the Impact of Climatic Variables on Malaria Transmission. Hacettepe J. Math. Stat..

[12] Abiodun G.J., Njabo K.Y., Witbooi P.J., Adeola A.M., Fuller T.L., Okosun K.O., Makinde O.S., Botai J.O. (2018). Exploring the Influence of Daily Climate Variables on Malaria Transmission and Abundance of *Anopheles Arabiensis* over Nkomazi Local Municipality, Mpumalanga Province, South Africa. J. Environ. Public Health.

[13] Craig M.H., Snow R.W., Le Sueur D. (1999). A Climate-Based Distribution Model of Malaria Transmission in Sub-Saharan Africa. Parasitol. Today.

[14] Hoshen M.B., Morse A.P. (2004). A Weather-Driven Model of Malaria Transmission. Malar. J..

[15] Briët O.J.T., Vounatsou P., Gunawardena D.M., Galappaththy G.N.L., Amerasinghe P.H. (2008). Models for Short Term Malaria Prediction in Sri Lanka. Malar. J..

[16] Wangdi K., Singhasivanon P., Silawan T., Lawpoolsri S., White N.J., Kaewkungwal J. (2010). Development of Temporal Modelling for Forecasting and Prediction of Malaria Infections Using Time-Series and ARIMAX Analyses: A Case Study in Endemic Districts of Bhutan. Malar. J..

[17] Anwar M.Y., Lewnard J.A., Parikh S., Pitzer V.E. (2016). Time Series Analysis of Malaria in Afghanistan: Using ARIMA Models to Predict Future Trends in Incidence. Malar. J..

[18] Arab A., Jackson M.C., Kongoli C. (2014). Modelling the Effects of Weather and Climate on Malaria Distributions in West Africa. Malar. J..

[19] Endo N., Eltahir E.A.B. (2016). Environmental Determinants of Malaria Transmission in African Villages. Malar. J..

[20] Box G., Jenkins G. (2008). Time Series Analysis: Forecasting and Control.

[21] Briët O.J.T., Amerasinghe P.H., Vounatsou P. (2013). Generalized Seasonal Autoregressive Integrated Moving Average Models for Count Data with Application to Malaria Time Series with Low Case Numbers. PLoS ONE.

[22] (2011). Statistics South Africa: Census Report.

[23] Van Buuren S., Groothuis-Oudshoorn K. (2011). Multivariate Imputation by Chained Equations in R. J. Stat. Softw..

[24] Buuren S., Groothuis-Oudshoorn K., Robitzsch A., Doove L., Jolani S. (2014). Multivariate Imputation by Chained Equations Date.

[25] Agresti A. (2007). An Introduction to Categorical Data Analysis.

[26] Afrane Y.A., Little T.J., Lawson B.W., Githeko A.K., Yan G. (2008). Deforestation and Vectorial Capacity of Anopheles Gambiae Giles Mosquitoes in Malaria Transmission, Kenya. Emerg. Infect. Dis..

[27] Beck-Johnson L.M., Nelson W.A., Paaijmans K.P., Read A.F., Thomas M.B., Bjørnstad O.N. (2013). The Effect of Temperature on Anopheles Mosquito Population Dynamics and the Potential for Malaria Transmission. PLoS ONE.

[28] Weiss D.J., Bhatt S., Mappin B., Van Boeckel T.P., Smith D.L., Hay S.I., Gething P.W. (2014). Air Temperature Suitability for Plasmodium Falciparum Malaria Transmission in Africa 2000–2012: A High-Resolution Spatiotemporal Prediction. Malar. J..

[29] Ljung G.M., Box G.E.P. (1978). On a Measure of Lack of Fit in Time Series Models. Biometrika.

[30] Pankratz A. (1991). Forecasting with Dynamic Regression Models.

[31] Zhou G., Minakawa N., Githeko A.K., Yan G. (2004). Association between Climate Variability and Malaria Epidemics in the East African Highlands. Proc. Natl. Acad. Sci. USA.

[32] Burke A., Dandalo L., Munhenga G., Dahan-Moss Y., Mbokazi F., Ngxongo S., Coetzee M., Koekemoer L., Brooke B. (2017). A New Malaria Vector Mosquito in South Africa. Sci. Rep..

[33] (2016). Mayoral/Portfolio Committee.

[34] (2018). Community Survey 2016: Provincial Profile: Limpopo.

[35] Vuong Q.H. (1989). Likelihood Ratio Tests for Model Selection and Non-Nested Hypotheses. Econometrica.

[36] Laneri K., Paul R.E., Tall A., Faye J., Diene-Sarr F., Sokhna C., Trape J.-F., Rodó X. (2015). Dynamical Malaria Models Reveal How Immunity Buffers Effect of Climate Variability. Proc. Natl. Acad. Sci. USA.

[37] Hay S.I., Rogers D.J., Shanks G.D., Myers M.F., Snow R.W. (2001). Malaria Early Warning in Kenya. Trends Parasitol..

[38] Zinszer K., Kigozi R., Charland K., Dorsey G., Brewer T.F., Brownstein J.S., Kamya M.R., Buckeridge D.L. (2015). Forecasting Malaria in a Highly Endemic Country Using Environmental and Clinical Predictors. Malar. J..

[39] Pascual M., Cazelles B., Bouma M.J., Chaves L.F., Koelle K. (2008). Shifting Patterns: Malaria Dynamics and Rainfall Variability in an African Highland. Proc. Biol. Sci..

[40] Kabanda T., Jury M. (1999). Inter-Annual Variability of Short Rains over Northern Tanzania. Clim. Res..

[41] Clark C.O., Webster P.J., Cole J.E. (2003). Interdecadal Variability of the Relationship between the Indian Ocean Zonal Mode and East African Coastal Rainfall Anomalies. J. Clim..

[42] Amekudzi L., Yamba E., Preko K., Asare E., Aryee J., Baidu M., Codjoe S., Amekudzi L.K., Yamba E.I., Preko K. (2015). Variabilities in Rainfall Onset, Cessation and Length of Rainy Season for the Various Agro-Ecological Zones of Ghana. Climate.

